# Regional disparities in major cancer incidence in Korea, 1999-2018

**DOI:** 10.4178/epih.e2023089

**Published:** 2023-10-12

**Authors:** Eun Hye Park, Mee Joo Kang, Kyu-Won Jung, Eun Hye Park, E Hwa Yun, Hye-Jin Kim, Hyun-Joo Kong, Chang Kyun Choi, Jeong-Soo Im, Hong Gwan Seo, The Community of Population-Based Regional Cancer Registries

**Affiliations:** 1 Korea Central Cancer Registry, National Cancer Center, Goyang, Korea; 2 Division of Cancer Registration and Surveillance, National Cancer Control Institute, National Cancer Center, Goyang, Korea; 3 Graduate School of Public Health, Seoul National University, Seoul, Korea; 4 National Cancer Control Institute, National Cancer Center, Goyang, Korea; 5 National Cancer Center Graduate School of Cancer Science and Policy, National Cancer Center, Goyang, Korea

**Keywords:** Neoplasms, Incidence, Korea, Epidemiology, Small-area analysis

## Abstract

**OBJECTIVES:**

This study investigated regional disparities in the incidence of 8 major cancers at the municipal level in Korea during 1999-2018 and evaluated the presence or absence of hot spots of cancer clusters during 2014-2018.

**METHODS:**

The Korea National Cancer Incidence Database was used. Age-standardized incidence rates were calculated by gender and region at the municipal level for 4 periods of 5 years and 8 cancer types. Regional disparities were calculated as both absolute and relative measures. The possibility of clusters was examined using global Moran’s I with a spatial weight matrix based on adjacency or distance.

**RESULTS:**

Regional disparities varied depending on cancer type and gender during the 20-year study period. For men, the regional disparities of stomach, colon and rectum, lung, and liver cancer declined, and those of thyroid and prostate cancer recently decreased, despite an overall increasing incidence. For women, regional disparities in stomach, colon and rectum, lung, liver, and cervical cancer declined, that of thyroid cancer recently decreased, despite an overall increasing incidence, and that of breast cancer steadily increased. In 2014-2018, breast cancer (I, 0.61; 95% confidence interval [CI], 0.53 to 0.70) showed a high probability of cancer clusters in women, and liver cancer (I, 0.48; 95% CI, 0.40 to 0.56) showed a high probability of cancer clusters in men.

**CONCLUSIONS:**

Disparities in cancer incidence that were not seen at the national level were discovered at the municipal level. These results could provide important directions for planning and implementing local cancer policies.

## INTRODUCTION

The incidence of cancer is rising globally, with its distribution varying significantly across different world regions [1]. The International Agency for Research on Cancer (IARC) has made global cancer statistics available through the Global Cancer Observatory, thereby facilitating comprehensive global cancer surveillance [2]. Indicators such as incidence, prevalence, mortality, and survival are utilized to monitor the burden of cancer. Among these, disparities in cancer incidence have been instrumental in identifying risk factors [2]. The Global Cancer Statistics 2020 report indicates that disparities in incidences between countries were nearly 5-fold for men and nearly 4-fold for women [1]. These disparities between countries are indicative of differences in exposure to risk factors and obstacles to high-quality cancer prevention and early detection [1]. Disparities within countries mirror those between countries, but they more distinctly underscore social inequalities based on race, gender, socioeconomic status, and geographical location [3]. Furthermore, disparities within countries had low possibility of differences based on the completeness or quality of the cancer registry [4].

Cancer epidemiology focuses on the study of cancer occurrence and distribution, which often exhibits various levels of difference and heterogeneity [5]. In epidemiology, disease distribution is typically described by person, time, and place [6]. However, in cancer epidemiology, the aspect of “place” has been relatively underemphasized compared to person and time [7]. The creation of a cancer incidence map is an important initial step in incorporating the concept of place in cancer epidemiology. Such a map can provide a visual overview of regional disparities in cancer, enabling the identification of new patterns and cancer clusters that may be challenging to discern in tabular form [7,8]. When assessing health disparities based on geographical location, it is important to evaluate both absolute and relative measures [9]. In the past, regional disparities in cancer incidence were primarily assessed as range differences (RDs) and range ratios (RRs) using minimum and maximum values. However, RDs and RRs could potentially overstate disparities based on these extreme values. To address this issue, several measures of disparities, such as between-group variance (BGV), are often presented concurrently [10].

In Korea, a population-based cancer registry for cancer surveillance was established in 2005 [11]. Nationwide cancer incidence increased from 1999 to 2012, then decreased from 2012 to 2015, with no significant changes observed since then [12]. Despite the overall decrease in cancer incidence, regional disparities persist [13]. According to municipal-level cancer incidence between 1999 and 2013, relative disparities between regions ranged from 2-fold to 12-fold, depending on the type of cancer [13]. The National Health Plan 2030 aims to improve health equity across regions, making it crucial to monitor and reduce these regional disparities [14]. Consequently, implementing measures to regularly monitor regional disparities is essential to achieving the goal of disparity reduction. This study aimed to uncover regional disparities in municipal-level cancer incidence from 1999 to 2018, using both absolute and relative measures, and to investigate the potential existence of cancer clusters during the period from 2014 to 2018.

## MATERIALS AND METHODS

### Data source

We utilized the Korean National Cancer Incidence Database, provided by the Korea Central Cancer Registry (KCCR), for the period between 1999 and 2018. The KCCR, a nationwide population-based cancer registry, was established by the Korean Ministry of Health and Welfare in 1980 [15]. Since 1999, the KCCR has been publishing nationwide cancer statistics [11], and starting from 2016, it has been providing municipal-level cancer incidence statistics every 5 years [13]. Our research concentrated on 8 primary types of cancer, classified according to their International Classification of Diseases, 10th edition (ICD-10) codes: stomach (C16), colon and rectum (C18-C20), lung (C33-C34), thyroid (C73), women breast (C50), liver (C22), prostate (C61), and cervix uteri (C53). These particular cancers were chosen based on their high incidence rates (thyroid, lung, colon and rectum, stomach, breast, prostate, and liver cancer) and their inclusion in the National Cancer Screening Program (stomach, liver, colon and rectum, breast, cervical, and lung cancer) [16].

We obtained mid-year population data at the municipal level from Statistics Korea [17]. To bolster statistical stability, we segmented the cancer incidence data into 4 periods (1999-2003, 2004-2008, 2009-2013, and 2014-2018), stratified by gender and region. We also established and categorized geographical locations in accordance with the classification of administrative regions by Statistics Korea, which were based on population and regional attributes. The number of municipalities (*si* [city], *gun* [county], *gu* [district]) included in the analysis fluctuates for each period due to alterations in administrative regions between 1999 and 2018. The count of municipalities was ascertained based on the initial year of each period. This was computed by dividing it into 245 municipalities for 1999-2003, 247 for 2004-2008, 249 for 2009-2013, and 252 for 2014-2018. Supplementary Material 1 offers more comprehensive information on administrative regions for 2014-2018. Detailed results of incidence calculated at the municipal level can be accessed via the Statistics Korea website (http://kosis.kr).

### Variables

We used age-standardized rates (ASRs) per 100,000 people to measure incidence. ASRs are defined as the weighted average of age-specific rates, where the weights correspond to the proportions of individuals in the respective age groups within a standard population [18]. In this study, the ASRs were standardized based on the mid-year Korean population for 2020. We quantified regional disparities among municipalities using both absolute and relative measures. Absolute disparity measures included the RD, BGV, and regional gap, while relative disparities were measured using the RR [19]. We determined the regional gap in cancer incidence by comparing the average ASR for the top 20% of municipalities with the average ASR for the bottom 20% of municipalities, as defined by the Korean National Health Plan 2030 [14]. Supplementary Material 2 offers a more comprehensive explanation of variable definitions and equations. The regions with the highest and lowest ASR by cancer type are detailed in Supplementary Material 3.

### Statistical analysis

Global Moran’s I was used to quantify the existence or non-existence of cancer hot spots, with the aim of identifying more cancer cases within specific geographic regions, taking into account the size and age of the population [20-22]. The spatial weight matrix was constructed by defining relationships with neighboring regions based on either adjacency or distance. In an adjacency-based spatial weight matrix, regions that share a common boundary are considered neighbors. Conversely, in a distance-based spatial weight matrix, regions within a certain distance are deemed neighbors. This was examined by incrementally increasing the distance from 5 km to 200 km until no regions remained without links to neighbors [23]. Moran’s I values range from -1 to 1. Positive values suggest the clustering of regions with similarly high or low values, while a value of I= 0 indicates no spatial autocorrelation (i.e., complete spatial randomness). Negative values, in contrast, suggest dissimilar values between neighboring regions, akin to a chessboard [24].

To illustrate regional disparities in incidence rates, we generated disease maps using R version 4.2.2 (R Foundation for Statistical Computing, Vienna, Austria). Each quintile interval encompassed an equal number of regions, which were depicted on the regional disease map. The fifth quintile, representing regions with the highest incidence, is indicated in the darkest shade. Statistical analysis was conducted using SAS version 9.3 (SAS Institute Inc., Cary, NC, USA) and RStudio version 2023.3.0.386 (R Foundation for Statistical Computing).

### Ethics statement

The Institutional Review Board of the National Cancer Center waived the requirement of ethics review for this research, as the study used anonymized data (IRB No. NCC2023-0135).

## RESULTS

### National and regional incidence of major cancers among Korean

**Figure 1.**
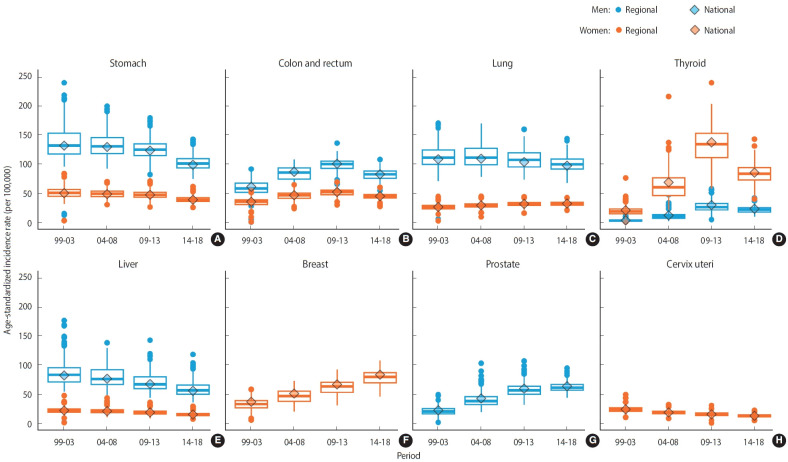
National and regional incidence of major cancers (A) stomach, (B) colon and rectum, (C) lung, (D) thyroid, (E) liver, (F) breast, (G) prostate, and (H) cervix uteri among Koreans, 1999-2018. The national incidence is represented by a diamond shape, while the regional incidence is depicted as a boxplot. The median of the regional incidence is marked by a line within the box. The box representing the interquartile range illustrates the middle 50% of regions, demonstrating the distance between the first and third quartiles (Q3-Q1). The numbers displayed in the graph represent age-standardized incidence rates per 100,000.

Figure 1 illustrates the national and regional incidence rates of major cancers among Koreans. In men, the national incidence rates of stomach, lung, and liver cancer steadily declined from 1999 to 2018, while the incidence of prostate cancer consistently risen. In women, the national incidence rates of stomach, liver, and cervical cancers also decreased during the 20-year study period, whereas the incidence rates of breast and lung cancer rose. The national incidence rates of colon and rectum cancer, as well as thyroid cancer, showed an upward trend over a span of 15 years in both men and women, but these rates decreased in the last 5 years of the study period. The regional incidence rates are represented in a boxplot, with the median values, indicated by a line within the box, mirroring the trend of national incidence. The interquartile range box illustrates the range between the first and third quartiles of regional incidence rates over time.

### Trends in regional disparities in major cancer incidence among Korean men

Table 1 illustrates the regional disparities in the incidence of major cancers among Korean men. The extent of these disparities varied based on the measurement method used. In 1999-2003 and 2004-2008, stomach cancer exhibited the largest regional disparities in RD, BGV, and regional gap, with the exception of RR. During 2009-2013, the largest regional disparities in incidence were observed for thyroid cancer when measured by RR, stomach cancer when measured by RD and regional gap, and lung cancer when measured by BGV. In 2014-2018, the most pronounced regional disparities in incidence were found for thyroid cancer when measured by RR, liver cancer when measured by RD, and lung cancer when measured by both BGV and regional gap (Table 1).

### Trends in regional disparities in major cancer incidence among Korean women

Table 2 shows the regional disparities in the incidence of major cancers among Korean women. In 1999-2003, the regional disparities in incidence were observed for thyroid cancer in RR, stomach cancer in RD and the regional gap, and breast cancer in BGV. Between 2004 and 2008, thyroid cancer exhibited the greatest regional disparities across all measures. In the periods from 2009 to 2013 and 2014 to 2018, thyroid cancer continued to show the most pronounced regional disparities in RD, BGV, and the regional gap, with the exception of RR (Table 2).

### Regional disparities in major cancer incidence during 2014-2018

**Figure 2.**
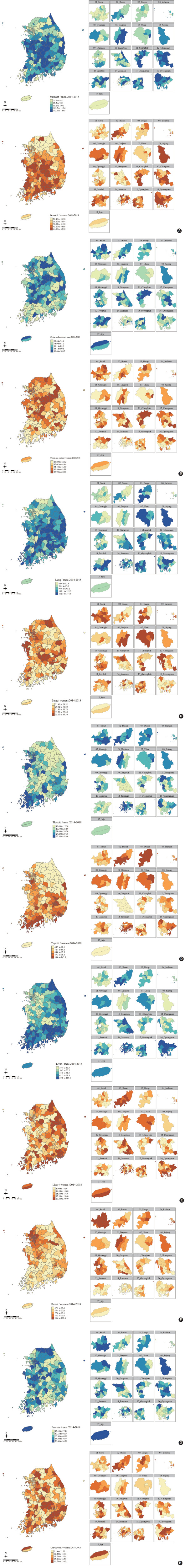
Municipal-level incidence of major cancers (A) stomach, (B) colon and rectum, (C) lung, (D) thyroid, (E) liver, (F) breast, (G) prostate, and (H) cervix uteri among Koreans in 2014-2018.

Figure 2 visualizes the regional incidence of major cancers, categorized by quantile, on a municipal-level map spanning all 17 provinces. For stomach cancer, regions with a high incidence were predominantly found in Chungnam and Gyeongbuk for men, and in Gyeongnam and Gyeongbuk for women (Figure 2A). For colon cancer and rectum cancer, regions with a high incidence were primarily situated in Gyeonggi and Seoul for men, and in Gyeonggi and Chungbuk for women (Figure 2B). For lung cancer, regions with a high incidence were largely located in Gyeongbuk and Gyeongnam for men, and in Gyeonggi and Seoul for women (Figure 2C). For thyroid cancer, regions with a high incidence were mainly found in Busan and Jeonnam for women, and in Gyeonggi and Jeonnam for men (Figure 2D). For liver cancer, regions with a high incidence were situated in Jeonnam and Gyeongnam for men, and in Gyeongnam and Jeonnam for women (Figure 2E). For breast cancer, regions with a high incidence were primarily located in Seoul and Gyeonggi for women (Figure 2F). For prostate cancer, regions with a high incidence were largely found in Seoul and Gyeonggi for men (Figure 2G). For cervical cancer, regions with a high incidence were mainly situated in Gyeongnam and Gyeonggi for women (Figure 2H). These findings highlight regions with a high likelihood of exposure to risk factors or high accessibility to cancer screening, depending on the type of cancer.

**Figure 3.**
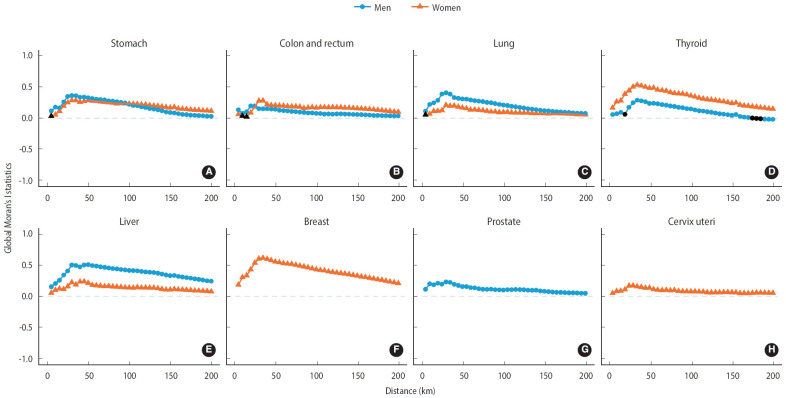
Distance-based global Moran’s I of major cancers (A) stomach, (B) colon and rectum, (C) lung, (D) thyroid, (E) liver, (F) breast, (G) prostate, and (H) cervix uteri incidence among Koreans in 2014-2018. All values are statistically significant, except for those shaded in black. I>0 indicates a clustering of areas with similar high or low values. I=0 denotes no spatial autocorrelation or complete spatial randomness. I<0 indicates neighboring areas that have dissimilar values, like a chessboard pattern.

In men, the largest regional gap were observed in the incidence rates of lung cancer (36.2 per 100,000; 95% CI, 14.3 to 58.2) and stomach cancer (36.1 per 100,000; 95% CI, 15.7 to 56.4). These disparities were calculated by determining the difference in average incidence between the highest and lowest quintiles (Table 1). For women, the greatest regional gap were found in the incidence rates of thyroid cancer (47.0 per 100,000; 95% CI, 26.8 to 67.1) and breast cancer (35.0 per 100,000; 95% CI, 16.5 to 53.6; Table 2). Table 3 presents spatial autocorrelation values based on adjacency and distance. Among men, liver cancer demonstrated the highest likelihood of cancer clusters, based on both adjacency (I, 0.48; 95% CI, 0.40 to 0.56) and distance (I, 0.51; 95% CI, 0.47; Figure 3). In women, breast cancer exhibited the highest likelihood of cancer clusters, both in terms of adjacency (I, 0.61; 95% CI, 0.53 to 0.70) and distance (I, 0.62; 95% CI, 0.55 to 0.69; Figure 3).

## DISCUSSION

This study revealed regional disparities in the incidence rates of 8 major cancers in Korea over a recent 2-decade period, examined at the municipal level. The incidence rates for each type of cancer displayed significant variation by both gender and region. The description of these regional disparities varied depending on whether absolute or relative measures were used. RR and RD, which utilized minimum and maximum values, exhibited substantial fluctuations over time. Notably, the case count in certain regions may have been underestimated, leading to an overstatement of regional disparities, particularly with the introduction of new administrative districts at the municipal level between 1999 and 2003. Conversely, the BGV and regional gap, as defined in the National Health Plan 2030, provided more reliable results for monitoring overall trends and statuses. The most significant regional disparity among women was found in thyroid cancer, followed by breast cancer and stomach cancer. For men, the largest disparities were seen in lung cancer, stomach cancer, and liver cancer. The areas suspected of being cancer hotspots at the municipal level were those with high incidences of breast and thyroid cancer in women, and liver, stomach, and lung cancer in men.

Regional disparities in cancer incidence reflect differences in risk prevalence and exposure, access to preventive measures and early detection, as well as healthcare utilization [3]. Thyroid cancer saw a significant rise in both incidence and regional disparities from 1999-2003 to 2009-2013. This was followed by a marked decrease after a report of overdiagnosis in 2014 [25]. Despite this, thyroid cancer still exhibited the highest regional disparity among women (Figure 1 and Table 2). The strong correlation between the proportion of cancer screenings and the regional incidence of thyroid cancer suggests that these disparities may be due to differences in healthcare utilization [25-27]. The rise in breast cancer incidence and disparities aligns with the global trend [1]. Globally, breast cancer is more prevalent in countries with a high Human Development Index. Similarly, in Korea, breast cancer is predominantly found in Seoul and other metropolitan areas. These disparities can be traced back to the prevalence of breast cancer risk factors. These include reproductive and hormonal risk factors such as early age at menarche and oral contraceptive use, as well as lifestyle risk factors like alcohol consumption, excess body weight, and physical inactivity. Differences in access to mammography screening also contribute to these disparities [28,29]. Prostate cancer follows a trend similar to that of breast cancer, with incidence known to increase with age in the population [30]. The cause of regional disparities in ASRs is not entirely clear, but it may be linked to the coverage of prostate-specific antigen testing [31].

The incidence of lung cancer in men has declined, yet it continues to exhibit a higher regional disparity and a potential for clusters. Furthermore, the incidence is still on the rise in women. It is well established that regional disparities in lung cancer are strongly correlated with regional smoking rates [32-34]. Occupational exposure to substances such as asbestos [35] and environmental exposure to factors like air pollution [36] are also known to be linked to lung cancer. The incidence of colon and rectum cancer saw an increase from 2009 to 2013, but has recently shown a decrease in both incidence and regional disparity. This shift can be attributed to the removal of precancerous lesions through colonoscopy and lifestyle improvements [37,38]. The incidence of stomach, liver, and cervical cancers has consistently shown reductions in both incidence and regional disparities. These reductions are believed to be the result of early screening, antiviral treatment for hepatitis, and *Helicobacter pylori* eradication treatment. The regional disparities have also been mitigated through the national immunization program and the national cancer screening program [39-42].

The high likelihood of cancer clusters indicates the existence of hot spots where cancer is densely concentrated in geographically proximate areas [20]. Liver cancer, stomach cancer, and lung cancer, despite seeming to have diminished regional disparities, are still highly likely to exhibit cancer clusters in men. This suggests that geographical location should be taken into account when monitoring regional disparities. In the case of liver cancer, defining the spatial weight matrix based on distance resulted in a slightly higher clustering possibility than when defining the spatial weight matrix based on adjacency. This implies that the scope of risk factors associated with cancer clusters is significantly larger than that of other cancers. It also underscores the need to reassess the spatial unit and the definition of neighboring regions, varying according to the suspected risk factors associated with each cancer cluster.

This study has several limitations. First, we selected municipal administrative districts as the spatial unit of analysis. This may have resulted in spatial misclassification because it is difficult to fully account for differences in the spatial and temporal range of exposure to risk factors by cancer type. Second, the spatial classification was based on the patient’s residence at the time of cancer diagnosis, which may not reflect the patient’s residence history and past exposures. Third, the modifiable areal unit problem may arise when the spatial unit is changed from the municipal level. Fourth, the global Moran’s I can only confirm the presence or absence of clusters—that is, it does not explain why cancer clusters occur. Furthermore, population heterogeneity could produce positive spatial autocorrelation, creating the illusion of meaningful clusters when they might simply be a statistical chance. Nevertheless, it is meaningful that monitoring the regional disparities in major cancer incidence at the municipal level can uncover new patterns and potential cancer clusters that were not detectable at the national level.

This study presents a detailed analysis of the incidence and regional disparities of 8 major types of cancer in Korea, broken down by gender and region. The findings from this study could serve as a preliminary draft for a more comprehensive analysis of specific types of cancer in regions with persistently high cancer incidence rates. Furthermore, this information could establish a foundation for the development of regional-specific cancer management policies in collaboration with local authorities.

## Figures and Tables

**Table 1.** Trends in incidence rates and regional disparities in the incidence of major cancers among Korean men

Cancer	Period	National incidence	Regional incidence			Measure of relative disparity	Measure of absolute disparity		
			Min	Median	Max	RR (95% CI)	RD (95% CI)	BGV (95% CI)	Regional gap^1^ (95% CI)
Stomach	1999-2003	132.6	13.1	133.1	239.9	18.3 (2.8, 33.8)	226.8 (193.4, 260.2)	398.5 (355.3, 441.8)	73.8 (41.2, 106.5)
	2004-2008	130.2	93.1	132.0	199.7	2.1 (1.6, 2.6)	106.6 (73.9, 139.3)	251.4 (220.0, 282.7)	55.8 (26.6, 85.1)
	2009-2013	124.2	71.4	125.8	179.6	2.5 (1.6, 3.5)	108.2 (71.4, 145.0)	177.8 (154.8, 200.8)	45.8 (21.4, 70.3)
	2014-2018	99.6	75.7	102.0	143.5	1.9 (1.4, 2.3)	67.8 (42.0, 93.6)	117.8 (103.2, 132.4)	36.1 (15.7, 56.4)
Colon and rectum	1999-2003	61.8	1.2	58.8	92.4	77.0 (-71.5, 225.5)	91.2 (71.6, 110.8)	115.1 (97.1, 133.2)	34.3 (15.2, 53.5)
	2004-2008	87.6	52.0	86.2	109.1	2.1 (1.3, 2.8)	57.1 (36.5, 77.7)	112.3 (94.4, 130.1)	34.7 (13.7, 55.7)
	2009-2013	101.4	54.9	100.8	136.9	2.5 (1.5, 3.5)	82.0 (46.5, 117.5)	73.9 (59.8, 88.0)	29.2 (7.1, 51.3)
	2014-2018	83.3	55.6	83.2	108.7	2.0 (1.3, 2.6)	53.1 (26.5, 79.7)	74.6 (63.5, 85.7)	25.9 (7.9, 43.9)
Lung	1999-2003	109.3	6.6	112.3	170.5	25.8 (-4.9, 56.6)	163.9 (137.4, 190.4)	267.8 (230.6, 305.0)	54.3 (25.7, 82.8)
	2004-2008	110.1	79.0	113.8	170.3	2.2 (1.6, 2.7)	91.3 (62.4, 120.2)	251.1 (218.5, 283.6)	51.9 (24.7, 79.1)
	2009-2013	104.4	74.3	108.9	160.2	2.2 (1.7, 2.6)	85.9 (56.1, 115.7)	178.4 (155.9, 200.9)	43.9 (18.8, 69.1)
	2014-2018	98.1	68.6	101.4	144.6	2.1 (1.3, 2.9)	76.0 (38.7, 113.3)	120.3 (105.0, 135.6)	36.2 (14.3, 58.2)
Thyroid	1999-2003	4.2	0.7	3.8	14.9	21.3 (-18.6, 61.1)	14.2 (10.4, 18.0)	3.3 (2.4, 4.2)	4.9 (0.2, 9.6)
	2004-2008	12.9	2.9	10.5	48.1	16.6 (-16.1, 49.2)	45.2 (37.3, 53.1)	39.9 (35.6, 44.3)	14.8 (8.1, 21.6)
	2009-2013	30.5	5.5	26.9	58.6	10.7 (2.1, 19.2)	53.1 (46.8, 59.4)	94.5 (86.9, 102.1)	24.8 (15.1, 34.6)
	2014-2018	24.1	10.6	22.5	42.6	4.0 (1.3, 6.7)	32.0 (23.4, 40.6)	27.4 (24.2, 30.6)	16.7 (6.5, 26.9)
Liver	1999-2003	83.6	3.4	85.7	177.9	52.3 (-28.6, 133.3)	174.5 (118.2, 230.8)	278.3 (248.0, 308.6)	56.5 (31.2, 81.8)
	2004-2008	78.0	49.9	77.7	139.7	2.8 (2.1, 3.5)	89.8 (67.1, 112.5)	219.7 (196.3, 243.2)	50.5 (27.3, 73.6)
	2009-2013	68.8	45.2	69.2	144.2	3.2 (2.1, 4.3)	99.0 (54.0, 144.0)	151.7 (135.7, 167.7)	40.6 (20.9, 60.3)
	2014-2018	57.1	37.4	58.8	119.6	3.2 (2.5, 3.9)	82.2 (59.6, 104.8)	95.3 (85.2, 105.3)	33.7 (17.1, 50.3)
Prostate	1999-2003	23.9	3.0	21.7	50.6	16.9 (-16.5, 50.2)	47.6 (36.4, 58.8)	59.1 (47.8, 70.3)	19.5 (6.4, 32.6)
	2004-2008	44.0	20.4	39.0	104.5	5.1 (3.1, 7.1)	84.1 (71.3, 96.9)	185.3 (163.4, 207.2)	31.4 (16.0, 46.8)
	2009-2013	60.9	33.2	57.4	108.4	3.3 (1.7, 4.8)	75.2 (56.2, 94.2)	144.8 (127.2, 162.4)	29.3 (12.6, 46.0)
	2014-2018	65.0	45.1	63.0	96.2	2.1 (1.7, 2.6)	51.1 (39.7, 62.5)	76.2 (65.2, 87.2)	23.9 (7.5, 40.3)

Min, minimum; Max, maximum; RR, range ratio; RD, range difference; BGV, between group variance; ASR, age-standardized rate.

1 The regional gap of cancer incidence between the top 20% ASR group and the bottom 20% ASR group according to the definition in the National Health Plan 2030; The Min values for 1999-2003 are very low because there were regions newly promoted to municipalities.

**Table 2.** Trends in incidence rates and regional disparities in the incidence of major cancers among Korean women

Cancer	Period	National incidence	Regional incidence			Measure of relative disparity	Measure of absolute disparity		
			Min	Median	Max	RR (95% CI)	RD (95% CI)	BGV (95% CI)	Regional gap^1^ (95% CI)
Stomach	1999-2003	50.9	3.6	51.3	84.7	23.5 (-22.5, 69.5)	81.1 (63.1, 99.1)	55.0 (46.7, 63.4)	27.2 (10.3, 44.2)
	2004-2008	49.3	31.1	49.9	70.7	2.3 (1.5, 3.1)	39.6 (26.2, 53.0)	37.6 (31.1, 44.2)	20.1 (5.1, 35.1)
	2009-2013	48.0	27.1	48.1	71.9	2.7 (1.6, 3.7)	44.8 (28.3, 61.3)	32.4 (27.1, 37.7)	19.0 (5.6, 32.4)
	2014-2018	39.6	26.4	39.7	62.1	2.4 (1.5, 3.2)	35.7 (20.9, 50.5)	19.2 (15.7, 22.6)	15.9 (2.7, 29.2)
Colon and rectum	1999-2003	36.0	1.7	35.8	55.7	32.8 (-33.3, 98.8)	54.0 (39.9, 68.1)	28.0 (22.8, 33.1)	18.2 (5.9, 30.5)
	2004-2008	47.5	24.5	46.8	65.3	2.7 (1.2, 4.1)	40.8 (17.3, 64.3)	31.1 (25.2, 37.0)	18.8 (4.9, 32.7)
	2009-2013	52.9	30.8	52.7	70.0	2.3 (1.4, 3.2)	39.2 (19.8, 58.6)	23.7 (18.9, 28.6)	17.9 (3.1, 32.6)
	2014-2018	45.7	28.2	45.7	60.9	2.2 (1.3, 3.0)	32.7 (11.9, 53.5)	15.2 (11.9, 18.6)	13.5 (0.8, 26.2)
Lung	1999-2003	27.1	3.3	27.0	45.3	13.7 (-5.6, 33.0)	42.0 (32.6, 51.4)	17.0 (13.2, 20.9)	13.4 (1.7, 25.1)
	2004-2008	30.0	10.1	29.7	45.6	4.5 (-0.7, 9.8)	35.5 (20.0, 51.0)	12.8 (9.6, 16.0)	12.3 (0.5, 24.2)
	2009-2013	32.4	16.6	32.1	44.8	2.7 (1.5, 3.9)	28.2 (16.6, 39.8)	10.6 (7.9, 13.3)	11.7 (0.7, 22.6)
	2014-2018	33.1	21.4	33.0	43.3	2.0 (1.1, 3.0)	21.9 (6.5, 37.3)	9.2 (6.9, 11.6)	10.4 (-0.3, 21.1)
Thyroid	1999-2003	21.6	1.6	19.3	77.1	48.2 (-43.8, 140.2)	75.5 (67.7, 83.3)	66.2 (58.8, 73.7)	18.7 (9.7, 27.7)
	2004-2008	69.6	22.5	60.3	216.3	9.6 (5.7, 13.6)	193.8 (179.2, 208.4)	644.2 (610.6, 677.9)	66.4 (49.1, 83.6)
	2009-2013	138.2	59.1	134.8	239.9	4.1 (3.2, 4.9)	180.8 (159.2, 202.4)	758.3 (719.4, 797.1)	89.4 (64.7, 114.1)
	2014-2018	86.2	40.9	84.2	143.8	3.5 (2.1, 5.0)	102.9 (72.3, 133.5)	213.2 (197.3, 229.1)	47.0 (26.8, 67.1)
Breast	1999-2003	38.2	6.9	33.7	59.7	8.7 (0.8, 16.5)	52.8 (44.7, 60.9)	72.6 (65.1, 80.0)	26.8 (15.9, 37.8)
	2004-2008	52.5	21.6	47.5	74.3	3.4 (1.8, 5.0)	52.7 (41.1, 64.3)	92.3 (83.2, 101.4)	31.1 (17.1, 45.2)
	2009-2013	68.1	32.1	64.2	93.9	2.9 (1.9, 4.0)	61.8 (48.7, 74.9)	105.8 (95.3, 116.2)	34.8 (18.2, 51.5)
	2014-2018	84.8	47.3	80.8	109.4	2.3 (1.7, 3.0)	62.1 (47.7, 76.5)	108.6 (97.4, 119.8)	35.0 (16.5, 53.6)
Liver	1999-2003	23.4	2.4	23.2	48.9	20.4 (-19.5, 60.2)	46.5 (32.7, 60.3)	18.5 (14.9, 22.2)	14.9 (3.7, 26.0)
	2004-2008	22.2	12.1	21.9	44.8	3.7 (0.7, 6.7)	32.7 (17.4, 48.0)	13.9 (11.0, 16.8)	13.2 (2.2, 24.2)
	2009-2013	20.0	10.0	19.9	37.3	3.7 (1.5, 5.9)	27.3 (16.6, 38.0)	12.5 (10.2, 14.8)	11.6 (2.3, 20.9)
	2014-2018	16.4	8.6	16.3	30.4	3.5 (0.4, 6.7)	21.8 (1.9, 41.7)	7.5 (6.1, 9.0)	9.6 (1.2, 18.0)
Cervix uteri	1999-2003	25.3	11.4	24.9	50.7	4.4 (1.1, 7.8)	39.3 (14.1, 64.5)	19.2 (15.7, 22.8)	15.2 (3.7, 26.7)
	2004-2008	20.0	9.6	20.0	33.6	3.5 (0.7, 6.3)	24.0 (5.8, 42.2)	9.2 (7.1, 11.3)	10.7 (0.5, 20.9)
	2009-2013	16.7	1.8	16.9	32.0	17.8 (-9.1, 44.7)	30.2 (8.8, 51.6)	7.9 (6.3, 9.4)	10.1 (0.8, 19.4)
	2014-2018	14.2	6.1	14.3	23.6	3.9 (-1.8, 9.6)	17.5 (4.9, 30.1)	5.7 (4.5, 6.9)	8.2 (-0.2, 16.5)

Min, minimum; Max, maximum; RR, range ratio; RD, range difference; BGV, between group variance; ASR, age-standardized rate.

1 The regional gap of cancer incidence between the top 20% ASR group and the bottom 20% ASR group according to the definition in the national health plan 2030; The Min values for 1999-2003 are very low because there were regions newly promoted to municipalities.

**Table 3.** Global Moran’s I^1^ according to adjacency and distance of major cancers among Koreans, 2014-2018

Cancer	Adjacency-based^2***^		Distance-based^3***^			
	Men	Women	km	Men	km	Women
Stomach	0.44 (0.35, 0.52)	0.33 (0.25, 0.41)	30	0.36 (0.29, 0.44)	35	0.29 (0.22, 0.36)
Colon and rectum	0.21 (0.13, 0.30)	0.24 (0.16, 0.32)	20	0.20 (0.08, 0.31)	35	0.28 (0.22, 0.35)
Lung	0.43 (0.34, 0.51)	0.22 (0.14, 0.31)	30	0.41 (0.33, 0.49)	30	0.21 (0.13, 0.29)
Thyroid	0.32 (0.24, 0.40)	0.56 (0.48, 0.64)	35	0.29 (0.22, 0.36)	35	0.54 (0.47, 0.61)
Liver	0.48 (0.40, 0.56)	0.20 (0.11, 0.28)	50	0.51 (0.47, 0.56)	45	0.24 (0.19, 0.29)
Breast	-	0.61 (0.53, 0.70)	-	-	35	0.62 (0.55, 0.69)
Prostate	0.29 (0.21, 0.37)	-	30	0.23 (0.16, 0.31)	-	-
Cervix uteri	-	0.15 (0.07, 0.23)	-	-	30	0.17 (0.10, 0.25)

Values are presented as Moran’s I (95% confidence interval).

1 I>0 indicates a clustering of areas with similar high or low values; I=0: denotes no spatial autocorrelation or complete spatial randomness; I<0 indicates neighboring areas that have dissimilar values, like a chessboard pattern.

2 Moran’s I calculated using an adjacency-based spatial weight matrix defining regions that share a line segment (or border) and a point (or vertex) as neighbors.

3 Moran’s I calculated using a distance-based spatial weight matrix defining regions as neighbors if the computed distance from the coordinates of their centroids falls within the base radius; We noted the distance criteria and the corresponding value when Moran’s I was the largest.

*** p<0.001.

## References

[1] Sung H, Ferlay J, Siegel RL, Laversanne M, Soerjomataram I, Jemal A (2021). Global cancer statistics 2020: GLOBOCAN estimates of incidence and mortality worldwide for 36 cancers in 185 countries. CA Cancer J Clin.

[2] Piñeros M, Znaor A, Mery L, Bray F (2017). A global cancer surveillance framework within noncommunicable disease surveillance: making the case for population-based cancer registries. Epidemiol Rev.

[3] Vaccarella S, Lortet-Tieulent J, Saracci R, Fidler MM, Conway DI, Vilahur N (2019). Reducing social inequalities in cancer: setting priorities for research. Reducing social inequalities in cancer: evidence and priorities for research.

[4] Bray F, Parkin DM (2009). Evaluation of data quality in the cancer registry: principles and methods. Part I: comparability, validity and timeliness. Eur J Cancer.

[5] dos Santos Silva I (1999). Cancer epidemiology: principles and methods.

[6] Porta M https://www.oxfordreference.com/display/10.1093/acref/9780199976720.001.0001/acref-9780199976720.

[7] Mack TM (2020). Cancers in the urban environment: how malignant diseases are caused and distributed among the diverse people and neighborhoods of a major global metropolis.

[8] Hansell AL, Beale LA, Ghosh RE, Fortunato L, Fecht D, Järup L (2014). The environment and health atlas for England and Wales.

[9] Oakes JM, Kaufman JS (2017). Methods in social epidemiology.

[10] Ahn J, Harper S, Yu M, Feuer EJ, Liu B, Luta G (2018). Variance estimation and confidence intervals for 11 commonly used health disparity measures. JCO Clin Cancer Inform.

[11] Shin HR, Won YJ, Jung KW, Kong HJ, Yim SH, Lee JK (2005). Nationwide cancer incidence in Korea, 1999~2001; first result using the national cancer incidence database. Cancer Res Treat.

[12] Kang MJ, Jung KW, Bang SH, Choi SH, Park EH, Yun EH (2023). Cancer statistics in Korea: incidence, mortality, survival, and prevalence in 2020. Cancer Res Treat.

[13] Won YJ, Jung KW, Oh CM, Park EH, Kong HJ, Lee DH (2018). Geographical variations and trends in major cancer incidences throughout Korea during 1999-2013. Cancer Res Treat.

[14] Oh Y (2021). The National Health Plan 2030: its purpose and directions of development. J Prev Med Public Health.

[15] Shin HR, Won YJ, Jung KW, Park JG, Ahn YO (2004). Cancer registration and statistics in Korea. J Korean Assoc Cancer Prev.

[16] Kim Y, Jun JK, Choi KS, Lee HY, Park EC (2011). Overview of the national cancer screening programme and the cancer screening status in Korea. Asian Pac J Cancer Prev.

[17] Korean Statistical Information Service https://kosis.kr/statHtml/statHtml.do?orgId=101&tblId=DT_1B040M5&conn_path=I2.

[18] International Agency for Research on Cancer https://publications.iarc.fr/Book-And-Report-Series/Iarc-Scientific-Publications/Cancer-Incidence-In-Five-Continents%C2%A0Volume-XI-2021.

[19] Harper S, Lynch J, National Cancer Institute (U.S.) (2005). Methods for measuring cancer disparities: a review using data relevant to healthy people 2010 cancer-related objectives.

[20] Goodman M, LaKind JS, Fagliano JA, Lash TL, Wiemels JL, Winn DM (2014). Cancer cluster investigations: review of the past and proposals for the future. Int J Environ Res Public Health.

[21] Thun MJ, Sinks T (2004). Understanding cancer clusters. CA Cancer J Clin.

[22] Moran PA (1950). Notes on continuous stochastic phenomena. Biometrika.

[23] Duncan EW, White NM, Mengersen K (2017). Spatial smoothing in Bayesian models: a comparison of weights matrix specifications and their impact on inference. Int J Health Geogr.

[24] Duncan DT, Kawachi I, Roux AV (2018). Neighborhoods and health.

[25] Ahn HS, Kim HJ, Welch HG (2014). Korea’s thyroid-cancer “epidemic”--screening and overdiagnosis. N Engl J Med.

[26] Ahn HS, Welch HG (2015). South Korea’s thyroid-cancer “epidemic”--turning the tide. N Engl J Med.

[27] LeClair K, Bell KJ, Furuya-Kanamori L, Doi SA, Francis DO, Davies L (2021). Evaluation of gender inequity in thyroid cancer diagnosis: differences by sex in US thyroid cancer incidence compared with a meta-analysis of subclinical thyroid cancer rates at autopsy. JAMA Intern Med.

[28] Nari F, Park J, Kim N, Kim DJ, Jun JK, Choi KS (2023). Impact of health disparities on national breast cancer screening participation rates in South Korea. Sci Rep.

[29] Kang SY, Lee SB, Kim YS, Kim Z, Kim HY, Kim HJ (2021). Breast cancer statistics in Korea, 2018. J Breast Cancer.

[30] Rawla P (2019). Epidemiology of prostate cancer. World J Oncol.

[31] Lee HY, Kim DK, Doo SW, Yang WJ, Song YS, Lee B (2020). Time trends for prostate cancer incidence from 2003 to 2013 in South Korea: an age-period-cohort analysis. Cancer Res Treat.

[32] Park JY, Jang SH (2016). Epidemiology of lung cancer in Korea: recent trends. Tuberc Respir Dis (Seoul).

[33] Thandra KC, Barsouk A, Saginala K, Aluru JS, Barsouk A (2021). Epidemiology of lung cancer. Contemp Oncol (Pozn).

[34] Underwood JM, Townsend JS, Tai E, Davis SP, Stewart SL, White A (2012). Racial and regional disparities in lung cancer incidence. Cancer.

[35] Kim HR (2009). Overview of asbestos issues in Korea. J Korean Med Sci.

[36] Moon DH, Kwon SO, Kim SY, Kim WJ (2020). Air pollution and incidence of lung cancer by histological type in Korean adults: a Korean National Health Insurance Service Health Examinee Cohort Study. Int J Environ Res Public Health.

[37] Shin A, Jang D, Choe S, Won YJ, Jung KW, Park JW (2019). Colorectal cancer epidemiology in Korea. J Korean Med Assoc.

[38] Jung KU, Kim HO, Kim H (2022). Epidemiology, risk factors, and prevention of colorectal cancer-an English version. J Anus Rectum Colon.

[39] Yeo Y, Gwack J, Kang S, Koo B, Jung SJ, Dhamala P (2013). Viral hepatitis and liver cancer in Korea: an epidemiological perspective. Asian Pac J Cancer Prev.

[40] Suh YS, Lee J, Woo H, Shin D, Kong SH, Lee HJ (2020). National cancer screening program for gastric cancer in Korea: nationwide treatment benefit and cost. Cancer.

[41] Kwon JW, Tchoe HJ, Lee J, Suh JK, Lee JH, Shin S (2020). The impact of national surveillance for liver cancer: results from real-world setting in Korea. Gut Liver.

[42] Ha HI, Chang HK, Park SJ, Lim J, Won YJ, Lim MC (2021). The incidence and survival of cervical, ovarian, and endometrial cancer in Korea, 1999-2017: Korea Central Cancer Registry. Obstet Gynecol Sci.

