# Supplementary material for: Regional disparities in major cancer incidence in Korea, 1999-2018
Source: Epidemiol Health. 2023 Oct 12;45:e2023089. doi: 10.4178/epih.e2023089 (PMC10867526; doi:10.4178/epih.e2023089)

Supplementary Table S1. Number of provinces and municipalities according to period

	1999-2003	2004-2008	2009-2013	2014-2018
Number of Provinces	16	16	16	17
Number of Municipals	245	247	249	252
01_Seoul	25	25	25	25
02_Busan	16	16	16	16
03_Daegu	8	8	8	8
04_Incheon	10	10	10	10
05_Gwangju	5	5	5	5
06_Daejeon	5	5	5	5
07_Ulsan	5	5	5	5
08_Sejong	-	-	-	1
09_Gyeonggi	39	41	44	44
10_Gangwon	18	18	18	18
11_Chungbuk	13	13	13	14
12_Chungnam	16	16	17	16
13_Jeonbuk	15	15	15	15
14_Jeonnam	22	22	22	22
15_Gyeongbuk	24	24	24	24
16_Gyeongnam	20	20	20	22
17_Jeju	4	4	2	2

The first year of each period is used as the reference year

Supplementary Table S2. Number of population according to period

Period	Population for 5 years by municipalities		
	Min	Median	Max
**1999-2003**	50,210	705,932	3,275,784
**2004-2008**	48,800	740,937	3,596,834
**2009-2013**	52,128	822,451	3,360,209
**2014-2018**	49,567	896,876	3,297,204

Min, minimum; Max, maximum;

Supplementary Table S3. Classification of administrative areas of provincial and municipal level in Korea during 2014–2018

Name of Provinces	Number	Name of municipalities
01_Seoul	1	Jongno-gu
	2	Jung-gu
	3	Yongsan-gu
	4	Seongdong-gu
	5	Gwangjin-gu
	6	Dongdaemun-gu
	7	Jungnang-gu
	8	Seongbuk-gu
	9	Gangbuk-gu
	10	Dobong-gu
	11	Nowon-gu
	12	Eunpyeong-gu
	13	Seodaemun-gu
	14	Mapo-gu
	15	Yangcheon-gu
	16	Gangseo-gu
	17	Guro-gu
	18	Geumcheon-gu
	19	Yeongdeungpo-gu
	20	Dongjak-gu
	21	Gwanak-gu
	22	Seocho-gu
	23	Gangnam-gu
	24	Songpa-gu
	25	Gangdong-gu
02_Busan	26	Jung-gu
	27	Seo-gu
	28	Dong-gu
	29	Yeongdo-gu
	30	Busanjin-gu
	31	Dongnae-gu
	32	Nam-gu
	33	Buk-gu
	34	Haeundae-gu
	35	Saha-gu
	36	Geumjeong-gu
	37	Gangseo-gu
	38	Yeonje-gu
	39	Suyeong-gu
	40	Sasang-gu
	41	Gijang-gun
03_Daegu	42	Jung-gu

	43	Dong-gu
	44	Seo-gu
	45	Nam-gu
	46	Buk-gu
	47	Suseong-gu
	48	Dalseo-gu
	49	Dalseong-gun
04_Incheon	50	Jung-gu
	51	Dong-gu
	52	Nam-gu
	53	Yeonsu-gu
	54	Namdong-gu
	55	Bupyeong-gu
	56	Gyeyang-gu
	57	Seo-gu
	58	Ganghwa-gun
	59	Ongjin-gun
05_Gwangju	60	Dong-gu
	61	Seo-gu
	62	Nam-gu
	63	Buk-gu
	64	Gwangsan-gu
06_Daejeon	65	Dong-gu
	66	Jung-gu
	67	Seo-gu
	68	Yuseong-gu
	69	Daedeok-gu
07_Ulsan	70	Jung-gu
	71	Nam-gu
	72	Dong-gu
	73	Buk-gu
	74	Ulju-gun
08_Sejong	75	Sejong-si
09_Gyeonggi	76	Jangan-gu, Suwon-si
	77	Gwonseon-gu, Suwon-si
	78	Paldal-gu, Suwon-si
	79	Yeongtong-gu, Suwon-si
	80	Sujeong-gu, Seongnam-si
	81	Jungwon-gu, Seongnam-si
	82	Bundang-gu, Seongnam-si
	83	Uijeongbu-si
	84	Manan-gu, Anyang-si
	85	Dongan-gu, Anyang-si
	86	Wonmi-gu, Bucheon-si
	87	Sosa-gu, Bucheon-si

	88	Ojeong-gu, Bucheon-si
	89	Gwangmyeong-si
	90	Pyeongtaek-si
	91	Dongducheon-si
	92	Sangnok-gu, Ansan-si
	93	Danwon-gu, Ansan-si
	94	Deogyang-gu, Goyang-si
	95	Ilsandong-gu, Goyang-si
	96	Ilsanseo-gu, Goyang-si
	97	Gwacheon-si
	98	Guri-si
	99	Namyangju-si
	100	Osan-si
	101	Siheung-si
	102	Gunpo-si
	103	Uiwang-si
	104	Hanam-si
	105	Cheoin-gu, Yongin-si
	106	Giheung-gu, Yongin-si
	107	Suji-gu, Yongin-si
	108	Paju-si
	109	Icheon-si
	110	Anseong-si
	111	Gimpo-si
	112	Hwaseong-si
	113	Gwangju-si
	114	Yangju-si
	115	Pocheon-si
	116	Yeoju-si
	117	Yeoncheon-gun
	118	Gapyeong-gun
	119	Yangpyeong-gun
	120	Chuncheon-si
	121	Wonju-si
	122	Gangneung-si
	123	Donghae-si
	124	Taebaek-si
	125	Sokcho-si
	126	Samcheok-si
	127	Hongcheon-gun
	128	Hoengseong-gun
	129	Yeongwol-gun
	130	Pyeongchang-gun
	131	Jeongseon-gun
	132	Cheorwon-gun

	133	Hwacheon-gun
	134	Yanggu-gun
	135	Inje-gun
	136	Goseong-gun
	137	Yangyang-gun
11_Chungbuk	138	Sangdang-gu, Cheongju-si
	139	Seowon-gu, Cheongju-si
	140	Heungdeok-gu, Cheongju-si
	141	Cheongwon-gu, Cheongju-si
	142	Chungju-si
	143	Jecheon-si
	144	Boeun-gun
	145	Okcheon-gun
	146	Yeongdong-gun
	147	Jeungpyeong-gun
	148	Jincheon-gun
	149	Goesan-gun
	150	Eumseong-gun
	151	Danyang-gun
12_Chungnam	152	Dongnam-gu, Cheonan-si
	153	Seobuk-gu, Cheonan-si
	154	Gongju-si
	155	Boryeong-si
	156	Asan-si
	157	Seosan-si
	158	Nonsan-si
	159	Gyeryong-si
	160	Dangjin-si
	161	Geumsan-gun
	162	Buyeo-gun
	163	Seocheon-gun
	164	Cheongyang-gun
	165	Hongseong-gun
	166	Yesan-gun
	167	Taean-gun
13_Jeonbuk	168	Wansan-gu, Jeonju-si
	169	Deokjin-gu, Jeonju-si
	170	Gunsan-si
	171	Iksan-si
	172	Jeongeup-si
	173	Namwon-si
	174	Gimje-si
	175	Wanju_Gun
	176	Jinan-gun
	177	Muju-gun

	178	Jangsu-gun
	179	Imsil-gun
	180	Sunchang-gun
	181	Gochang-gun
	182	Buan-gun
14_Jeonnam	183	Mokpo-si
	184	Yeosu-si
	185	Suncheon-si
	186	Naju-si
	187	Gwangyang-si
	188	Damyang-gun
	189	Gokseong-gun
	190	Gurye-gun
	191	Goheung-gun
	192	Boseong-gun
	193	Hwasun-gun
	194	Jangheung-gun
	195	Gangjin-gun
	196	Haenam-gun
	197	Yeongam-gun
	198	Muan-gun
	199	Hampyeong-gun
	200	Yeonggwang-gun
	201	Jangseong-gun
	202	Wando-gun
	203	Jindo-gun
	204	Sinan-gun
15_Gyeongbuk	205	Nam-gu, Pohang-si
	206	Buk-gu, Pohang-si
	207	Gyeongju-si
	208	Gimcheon-si
	209	Andong-si
	210	Gumi-si
	211	Yeongju-si
	212	Yeongcheon-si
	213	Sangju-si
	214	Mungyeong_si
	215	Gyeongsan-si
	216	Gunwi-gun
	217	Uiseong-gun
	218	Cheongsong-gun
	219	Yeongyang-gun
	220	Yeongdeok-gun
	221	Cheongdo-gun
	222	Goryeong-gun

	223	Seongju-gun
	224	Chilgok-gun
	225	Yecheon-gun
	226	Bonghwa-gun
	227	Uljin-gun
	228	Ulleung-gun
16_Gyeongnam	229	Uichang-gu, Changwon-si
	230	Seongsan-gu, Changwon-si
	231	Masanhappo-gu, Changwon-si
	232	Masanhoewon-gu, Changwon-si
	233	Jinhae-gu, Changwon-si
	234	Jinju-si
	235	Tongyeong-si
	236	Sacheon-si
	237	Gimhae-si
	238	Miryang-si
	239	Geoje-si
	240	Yangsan-si
	241	Uiryeong-gun
	242	Haman-gun
	243	Changnyeong-gun
	244	Goseong-gun
	245	Namhae-gun
	246	Hadong-gun
	247	Sancheong-gun
	248	Hamyang-gun
	249	Geochang-gun
	250	Hapcheon-gun
17_Jeju	251	Jeju-si
	252	Seogwipo-si

Supplementary figure S1. Municipal-level map according to 17 provinces in Korea during 2014–2018

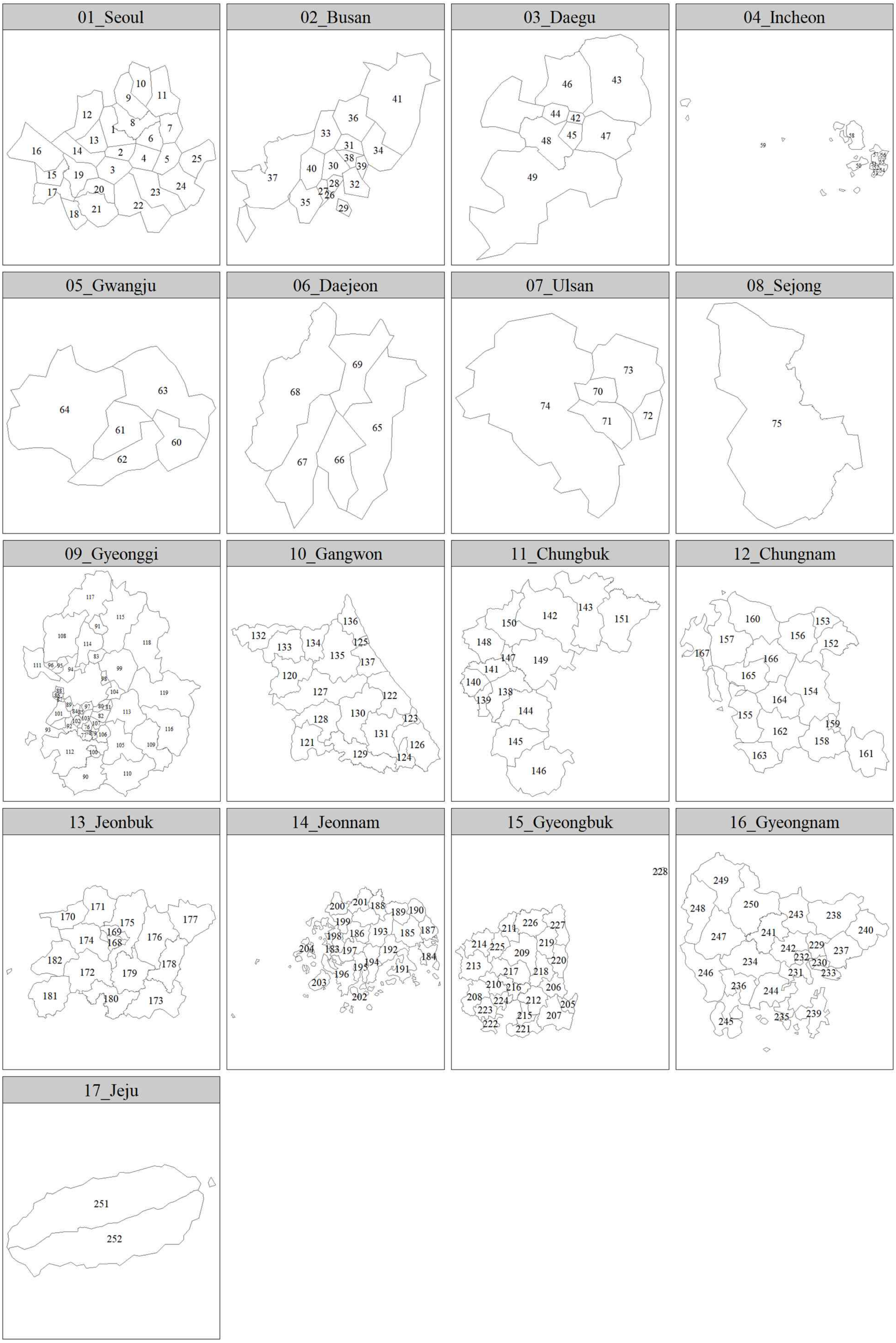

Supplement: Supplementary Material 1. [file epih-45-e2023089-Supplementary-1.pdf]
