# Supplementary material for: Regional disparities in major cancer incidence in Korea, 1999-2018
Source: Epidemiol Health. 2023 Oct 12;45:e2023089. doi: 10.4178/epih.e2023089 (PMC10867526; doi:10.4178/epih.e2023089)
Supplement: Supplementary Material 2. [file epih-45-e2023089-Supplementary-2.pdf]

Supplementary Appendix A: definition of measures

1. Age-standardized rates

$$ASR = \frac{\sum r_i w_i}{\sum w_i} \times 100{,}000$$

r_i : age-specific rate in the ith age class

w_i: the population present in the ith age class of the standard population

2. Range difference (RD)

$$\widehat{RD} = ASR_{max} - ASR_{min}$$

ASR_{max}: Age-standardized rates of the highest municipal

ASR_{min}: Age-standardized rates of the lowest municipal

$$var(RD) = var(Y_a - Y_b) = \sigma_a^2 + \sigma_b^2$$

$$Var\left(\widehat{RD}\right) = Var\left(ASR_{max} - ASR_{min}\right) = \sigma_{max}^2 + \sigma_{min}^2$$

3. Regional gap

Regional gap = Average age-standardized rates for top 20% municipalities - Average age-standardized rates for bottom 20% municipalities

$$Var\left(Regional\ \ gap\right) = Var\left(Mean(ASR_{top\ 20\%}) - Mean(ASR_{bottom\ \ 20\%})\right)$$

$$= \sigma_{top\ 20\%}^2 + \sigma_{bottom\ \ 20\%}^2$$

$$\sigma_{top\ 20\%}^2 = \frac{\sum \sigma_i}{n},\ \sigma_{bottom\ \ 20\%}^2 = \frac{\sum \sigma_i}{m}$$

4. Between-group variance (BGV)

$$\widehat{BGV} = \sum_{j=1}^{J} p_j (ASR_j - \mu)^2$$

ASR_j: Age-standardized rates of municipal j

p_j: Relative population size of municipal j, $\sum_{j=1}^{J} p_j = 1$

μ: Average age-standardized rates of the population, $\mu = \sum_{j=1}^{J} p_j y_j$

$$Var(\widehat{BGV}) = 4\sum_{j=1}^{J} p_j^2 \hat{\sigma}_j^{\,2} (ASR_j - \mu)^2 + 2\left[S_2^2 - S_4 + \sum p_j^2 (1-p_j)^2 \hat{\sigma}_j^4\right]$$

$$S_4 = \sum p_j^4 \, \hat{\sigma}_j^{\,4}, \quad S_2 = \sum p_j^2 \, \hat{\sigma}_j^{\,2},$$

5. Range ratio (RR)

$$\widehat{RR} = \frac{ASR_{max}}{ASR_{min}}$$

ASR_{max}: Age-standardized rates of the highest municipal

ASR_{min}: Health status of the worst group

$$Var(\widehat{RR}) = Var(ASR_{max}/ASR_{min}) = \frac{\sigma_{max}^2}{ASR_{min}^2} + \frac{ASR_{max}^2 \sigma_{min}^2}{ASR_{min}^4}$$

4. Moran's I

$$I = \frac{N \sum_a \sum_b W_{ab} (\mathrm{ASR_a} - \overline{\mathrm{ASR}})(\mathrm{ASR_b} - \overline{\mathrm{ASR}})}{(\sum_a \sum_b W_{ab}) \sum_a (\mathrm{ASR_a} - \overline{\mathrm{ASR}})^2}$$

W_{ab} : an $N \times N$ spatial weight matrix that measures the closeness between area a and its neighbor b.

adjacency-based spatial weight matrix $W_{ab} = \begin{cases} 1, & if \ area \ a \ and \ b \ are \ adjacent \\ 0, & otherwise \end{cases}$

Distance-based spatial weight matrix $W_{ab} =$
$\begin{cases} 1, & if \ d_{ab} < d \ for \ some \ fixed \ distance \ d \\ 0, & otherwise \end{cases}$
