# Supplementary material for: Regional disparities in major cancer incidence in Korea, 1999-2018
Source: Epidemiol Health. 2023 Oct 12;45:e2023089. doi: 10.4178/epih.e2023089 (PMC10867526; doi:10.4178/epih.e2023089)
Supplement: Supplementary Material 3. [file epih-45-e2023089-Supplementary-3.pdf]

Supplementary Table S4. Municipalities with the highest and lowest incidences of major cancer among Korean men

Cancer	Period	Highest incidence		Lowest incidence	
		Province	Municipality	Province	Municipality
**Stomach**	**1999-2003**	11_Chungbuk	Goesan-gun	11_Chungbuk	Jeungpyeong-gun
	**2004-2008**	15_Gyeongbuk	Yeongdeok-gun	09_Gyeonggi	Dongducheon-si
	**2009-2013**	12_Chungnam	Yeongi-gun	10_Gangwon	Yanggu-gun
	**2014-2018**	16_Gyeongnam	Sancheong-gun	14_Jeonnam	Wando-gun
**Colon and rectum**	**1999-2003**	11_Chungbuk	Goesan-gun	12_Chungnam	Gyeryong-si
	**2004-2008**	01_Seoul	Mapo-gu	13_Jeonbuk	Jangsu-gun
	**2009-2013**	11_Chungbuk	Jeungpyeong-gun	13_Jeonbuk	Jangsu-gun
	**2014-2018**	15_Gyeongbuk	Cheongsong-gun	09_Gyeonggi	Gwacheon-si
**Lung**	**1999-2003**	14_Jeonnam	Yeonggwang-gun	11_Chungbuk	Jeungpyeong-gun
	**2004-2008**	14_Jeonnam	Hwasun-gun	17_Jeju	Seogwipo-si
	**2009-2013**	15_Gyeongbuk	Gunwi-gun	09_Gyeonggi	Ilsandong-gu, Goyang-si
	**2014-2018**	15_Gyeongbuk	Yeongyang-gun	12_Chungnam	Gyeryong-si
**Thyroid**	**1999-2003**	14_Jeonnam	Yeosu-si	11_Chungbuk	Boeun-gun
	**2004-2008**	14_Jeonnam	Yeosu-si	10_Gangwon	Yanggu-gun
	**2009-2013**	01_Seoul	Gangnam-gu	10_Gangwon	Hoengseong-gun
	**2014-2018**	14_Jeonnam	Yeosu-si	12_Chungnam	Cheongyang-gun
**Liver**	**1999-2003**	15_Gyeongbuk	Ulleung-gun	11_Chungbuk	Jeungpyeong-gun
	**2004-2008**	16_Gyeongnam	Changnyeong-gun	04_Incheon	Ganghwa-gun
	**2009-2013**	15_Gyeongbuk	Ulleung-gun	09_Gyeonggi	Giheung-gu, Yongin-si
	**2014-2018**	14_Jeonnam	Jindo-gun	09_Gyeonggi	Bundang-gu, Seongnam-si
**Prostate**	**1999-2003**	01_Seoul	Seocho-gu	11_Chungbuk	Jeungpyeong-gun
	**2004-2008**	09_Gyeonggi	Bundang-gu, Seongnam-si	15_Gyeongbuk	Yecheon-gun
	**2009-2013**	09_Gyeonggi	Suji-gu, Yongin-si	11_Chungbuk	Jeungpyeong-gun
	**2014-2018**	01_Seoul	Seocho-gu	16_Gyeongnam	Sacheon-si

Jeungpyeong-gun and Gyeryong-si were promoted from branch office to municipality in 2003.

Supplementary Table S5. Municipalities with the highest and lowest incidences of major cancer among Korean women

Cancer	Period	Highest incidence		Lowest incidence	
		Province	Municipality	Province	Municipality
Stomach	1999-2003	11_Chungbuk	Goesan-gun	12_Chungnam	Gyeryong-si
	2004-2008	15_Gyeongbuk	Gimcheon-si	10_Gangwon	Hoengseong-gun
	2009-2013	15_Gyeongbuk	Uljin-gun	10_Gangwon	Cheorwon-gun
	2014-2018	14_Jeonnam	Boseong-gun	09_Gyeonggi	Gapyeong-gun
Colon and rectum	1999-2003	11_Chungbuk	Goesan-gun	11_Chungbuk	Jeungpyeong-gun
	2004-2008	11_Chungbuk	Jeungpyeong-gun	10_Gangwon	Yangyang-gun
	2009-2013	12_Chungnam	Cheongyang-gun	10_Gangwon	Cheorwon-gun
	2014-2018	10_Gangwon	Yanggu-gun	14_Jeonnam	Goheung-gun
Lung	1999-2003	15_Gyeongbuk	Yeongcheon-si	11_Chungbuk	Jeungpyeong-gun
	2004-2008	15_Gyeongbuk	Cheongdo-gun	15_Gyeongbuk	Ulleung-gun
	2009-2013	16_Gyeongnam	Goseong-gun	14_Jeonnam	Gangjin-gun
	2014-2018	11_Chungbuk	Jeungpyeong-gun	12_Chungnam	Cheongyang-gun
Thyroid	1999-2003	14_Jeonnam	Yeosu-si	11_Chungbuk	Jeungpyeong-gun
	2004-2008	14_Jeonnam	Yeosu-si	10_Gangwon	Yeongwol-gun
	2009-2013	14_Jeonnam	Gwangyang-si	10_Gangwon	Samcheok-si
	2014-2018	14_Jeonnam	Gangjin-gun	10_Gangwon	Yangyang-gun
Breast	1999-2003	01_Seoul	Gangnam-gu	11_Chungbuk	Jeungpyeong-gun
	2004-2008	09_Gyeonggi	Bundang-gu, Seongnam-si	14_Jeonnam	Hampyeong-gun
	2009-2013	01_Seoul	Seocho-gu	10_Gangwon	Jeongseon-gun
	2014-2018	01_Seoul	Gangnam-gu	10_Gangwon	Pyeongchang-gun
Liver	1999-2003	02_Busan	Gangseo-gu	11_Chungbuk	Jeungpyeong-gun
	2004-2008	14_Jeonnam	Jindo-gun	11_Chungbuk	Jeungpyeong-gun
	2009-2013	16_Gyeongnam	Namhae-gun	12_Chungnam	Cheongyang-gun
	2014-2018	15_Gyeongbuk	Ulleung-gun	15_Gyeongbuk	Gunwi-gun
Cervix	1999-2003	04_Incheon	Ongjin-gun	16_Gyeongnam	Hamyang-gun
	2004-2008	10_Gangwon	Yanggu-gun	16_Gyeongnam	Hamyang-gun
	2009-2013	15_Gyeongbuk	Ulleung-gun	14_Jeonnam	Jindo-gun
	2014-2018	13_Jeonbuk	Gochang-gun	15_Gyeongbuk	Ulleung-gun

Jeungpyeong-gun and Gyeryong-si were promoted from branch office to municipality in 2003.
